# Clearance rates of sand-burrowed and laterally pressed unburrowed Pismo clam *Tivela stultorum* (Mawe 1823) in a laboratory open-flow system

**DOI:** 10.1242/bio.060268

**Published:** 2024-03-28

**Authors:** Enrique Zepeda, Zaul Garcia-Esquivel, Marco A. González-Gómez, Fernando Díaz, Sheila Castellanos-Martinez

**Affiliations:** ^1^ Instituto de Investigaciones Oceanológicas, Universidad Autónoma de Baja California, Carretera Ensenada-Tijuana No. 3917, Fraccionamiento Playitas, 22860, Ensenada, Baja California, Mexico; ^2^ Departamento de Recursos del Mar, CINVESTAV, Mérida, Yucatán, Mexico; ^3^ Departamento de Biotecnología Marina, Centro de Investigación Científica y de Educación Superior de Ensenada (CICESE), Carretera Ensenada-Tijuana # 3918, Ensenada, Baja California, Mexico

**Keywords:** Pismo clam, Clearance rates, Feeding rates, *Tivela stultorum*, Burrowing

## Abstract

Pismo clam extraction is currently banned in Mexico to help the recovery of natural populations. Thus, the primary objective of this study was to gain insight on its basic biology and husbandry protocols. Growth and clearance rate (CR) of sand-burrowed and sediment-free, laterally pressed adult Pismo clams were quantified in the laboratory as a function of burrowing condition, flow, temperature, and microalgal concentration using open-flow chambers. After 40 days, clams remained healthy regardless of burrowing condition and showed a hyperbolic CR response pattern to increased flow, with CR directly proportional to flows lower than 1000 ml min^−1^. Maximal asymptotic CR values (300 to 400 ml min^−1^ org^−1^) were observed from 1000 to 2000 ml min^−1^. No significant CR differences were observed between burrowed and laterally pressed clams, yet microalgal concentration effects were detected, with constant maximal CRs of ∼250 ml min^−1^ in the range of 50 to 200 cells µl^−1^ and decline at higher concentrations. Maintenance protocols of laterally pressed organisms were validated in the laboratory with both weight and CR data. To our knowledge, this is the first study providing whole-body physiological data translated into effective husbandry protocols for Pismo clams. This approach represents a fresh perspective to traditional research areas, opening the possibility for continued experimentation under controlled conditions.

## Teaser

**Summary:** A novel method for maintaining healthy sediment-free Pismo clams, *Tivela stultorum*, in the laboratory was validated with whole-body physiological data under various environmental conditions.

## INTRODUCTION

The clearance rate of bivalve molluscs (CR, ml min^−1^) is a sensitive physiological variable defined as the rate at which a volume of water is cleared from 100% of their suspended particles, which usually happens with particle sizes ranging from 4–10 µm in many bivalves (Møhlenberg and Riisgård, 1978; Shumway et al., 1985; Cranford et al., 2011). A significant portion of the existing literature on CR relies on data gathered from undisturbed organisms positioned in incubation chambers with a specific cell concentration, such that changes in cell counts over time are used to calculate CR using the Coughlan (1969) equation. Another method using flow-through chambers was later incorporated in field and laboratory studies. The equations applied to estimate CR were based not only in the flow but also on the shape of the chamber, water path from the inflow to the outflow, and the organism position inside the chamber (e.g. Beiras et al., 1993; Filgueira et al., 2009; Larsen and Riisgård, 2012). In any case, the open-flow approach allows longer incubation times, continuous observations of experimental organisms and averaged CR values computed over several discrete recording periods.

Bivalve molluscs show high plasticity in their CR and its magnitude varies as a function of size, species and particle size (see Shumway et al., 1985; Newell et al., 1989; Ward and Shumway, 2004; MacDonald et al., 2006). Other environmental variables such as light, temperature (e.g. Hills et al., 2020; Pouil et al., 2021; Kamermans and Saurel, 2022), salinity (e.g. McFarland et al., 2013) and food quality/quantity (e.g. Bacon et al., 1998; Sejr et al., 2004; Filgueira et al., 2009; Pouil et al., 2021) play significant roles in shaping CRs. In particular, the effects of temperature and food quantity/quality are the most intensively documented. In this regard, CR as many other whole-body physiological variables show an enzyme-like pattern with respect to temperature, rising as temperatures increase and declining or stopping when approaching the lethal thermal limit (e.g. Petersen et al., 2003; Lim et al., 2008; Shin et al., 2009; Kamermans and Saurel, 2022). Marine bivalves are also capable of partially regulating their CR as a function of food concentration in order to reach and maintain maximal ingestion rates (Foster-Smith, 1975; Winter, 1978). Thus, when organisms feed on pure algal diets with low cell concentrations, they rapidly reach maximal CRs, which subsequently decrease exponentially as concentrations rise, while ingestion rates remain relatively constant (see Winter, 1978, MacDonald et al., 2006; Pascoe et al., 2009).

Bivalves feeding on high concentrations of pure microalgal may produce pseudofeces (e.g. Shumway et al., 1985) but to date there is still no consensus on threshold concentrations that triggers such production. Threshold concentration of 75 cells µl^−1^ was reported for the cockle *Cerastoderma edule* and the venerid *Venerupsis pullastra* feeding on *Isochrysis galbana*, while blue mussels (*Mytilus edulis*) required one third of such concentration to produced pseudofeces (Foster-Smith, 1975). Gatenby et al. (2013) also reported the production of pseudofeces by freshwater mussels, *Villosa iris*, at concentrations above 1 mg l^−1^(∼75 cells µl^−1^) of *Neochloris oleoabundans*. Conversely, Palmer (1980) reported no pseudofeces production in juvenile of the scallop *Argopecten irradians concentricus* or the oyster *Crassostrea virginica* when exposed to experimental concentrations of 3 mg l^−1^ of *Thalassiosira pseudonana* (∼100 cells µl), *I*. *galbana* (∼100 cells µl) or *Dunaliella tertiolecta* (Palmer, 1980). When bivalves are under more realistic environmental conditions they may not only modify their pumping activity (CR) but also the production of pseudofeces because the amount of ingested food depends on the total load and organic content of seston (MacDonald et al., 2006). There is abundant quantitative literature on this behavior showing that threshold seston concentrations for pseudofeces production vary among bivalve groups (reviewed by Bacon et al., 1998; MacDonald et al., 2006; Cranford et al., 2011). Thus, it is not surprising to observe clearance rates ranging from 1 to 10 L g^−1^ h^−1^ within a single species such as *Mytilus edulis* (reviewed by Cranford et al., 2011), and CRs ranging from 0.03 to 3.2 L g^−1^ h^−1^ in other venerid clam species (Zhuang and Wang, 2004; Chien and Hsu, 2006; Moschino and Marin, 2006; Lim et al., 2008; Shin et al., 2009; Metaxatos and Ignitiades, 2011).

The practical utility of bivalve CRs lies in their immediate response to shifting environmental or experimental conditions. It reflects changes in the metabolic state of an organism, especially when food acquisition is compromised (Beiras et al., 1993). For this reason CR is widely used in mussels as sentinels to assess the degree of environmental pollution in coastal waters (Beyer et al., 2017 and references therein) including aromatic hydrocarbons (Toro et al., 2003), toxicants (Salerno et al., 2018), and microplastics (Harris and Carrington, 2020). From an aquacultural perspective, CR can be used to gain information on the organism's response to current husbandry practices of economically important species. Data acquired to quantify CR is also commonly used to estimate the amount of food demanded by larvae (Gerdes, 1983; Pérez-Camacho et al., 1994; Doroudi et al., 2003; Ferreira-Arrieta et al., 2015; Angel-Dapa et al., 2021; Nava-Gómez et al., 2022), postlarvae (Gerdes, 1983; Aya et al., 2013; Nava-Gómez et al., 2022) and adults of bivalves (e. g. García-Esquivel et al., 2013; Le et al., 2017), which may eventually result in better aquaculture practices in the hatcheries. Therefore, the information gained with CR not only may be used to better understand the ecological adaptation of molluscan bivalves to their surrounding environment, but also to produce basic information on commercially important species whose fishery is under pressure and needs alternative options for restocking efforts and/or aquaculture production.

The Pismo clam, *Tivela stultorum*, is a temperate-subtropical species from the Veneridae family whose distribution ranges from Monterrey Bay, CA, USA to Punta San Juanico, Mexico on the west Pacific coast (Hall et al., 1974). This short-siphoned species live burrowed in shallow sandy sediments typically exposed to the surf along the intertidal-subtidal zone (Coe, 1947). Their lateral movement on the beach detected by GPS-enabled radiofrequency scanner is limited but takes place when the sediment is moved by heavy wave action and necessarily requires the ability of Pismo clams to unburrow and rebury (CDFW, 2022) as well as to open and close their heavy and thick valves during this process. Harvested Pismo clams can withstand several days with their valves closed outside the water, yet short- and long-term effects of keeping them unburrowed underwater is unknown. This fact is especially important from an aquaculture standpoint since keeping adults for conditioning in the laboratory requires that they stay healthy and competent for performing any physiological task.

The Mexican Pismo clam is a commercially exploited species whose catch decreased from ∼1500 tons (t) live weight in 1978–1980 (Searcy-Berna and Juárez-Romero, 1991) to ∼100 t in 1994 and 2003 (source: Secretaría de Fomento Agropecuario de Baja California, México). Extraction of Pismo clams has been currently banned in Mexico from October to March in an attempt to help the recovery of natural populations. Recent government management plans identified the need for developing seed production protocols in the laboratory as an alternative for restocking natural areas or for aquaculture purposes (SEMARNAT, 2022), something that was already suggested a century ago as an alternative to relieve the pressure of the fishery on natural Pismo clam populations of California (Weymouth, 1922). Unfortunately, since early field reports on growth, abundance and spawning habits of the species (e.g. Herrington, 1929; Coe, 1947), most of the studies carried out later have continued focusing on population structure and distribution (Marquardt et al., 2023; Greene, 2015), growth (Marquardt et al., 2022; Hall et al., 1974; Juárez-Romero and Searcy-Bernal., 1994), and reproductive aspects (Marquardt et al., 2022). Little is known about other biological aspects of the species including its physiology and husbandry even though there is valuable information already published on tissue biochemical content of adults (Giese et al., 1967), heart contraction (Flores et al., 2017), induction to oocyte maturation (Alvarado-Alvarez et al., 1996), fecundity and preliminary observations of laboratory-produced larvae (Pattison, 2001).

The primary objective of the present study is to gain insight on the basic Pismo clam biology and requirements for keeping them healthy in the laboratory. Given the CR’s importance as a whole-body physiological indicator, this variable and live weight were used as tools for assessing the overall health of Pismo clams and their response to lateral shell pressing force as an alternative for keeping them sediment-free in the laboratory. The effect of flow, temperature and microalgal concentration on CR were also determined for the first time in this species.

## RESULTS

### Growth

Clams kept in the main recirculating system had an initial mean size and live weight of 131±2.2 mm, 463±21.7 g (burrowed) and 127±4.4 mm, 432±37.9 g (laterally pressed) that were not statistically different (two-tailed *t*-test *P*>0.05 for both variables). Both groups showed the same temporal pattern, with a slight increase of their mean live weight during the first 10 days, and no significant changes thereafter (Fig. 3).

**Fig. 1.**
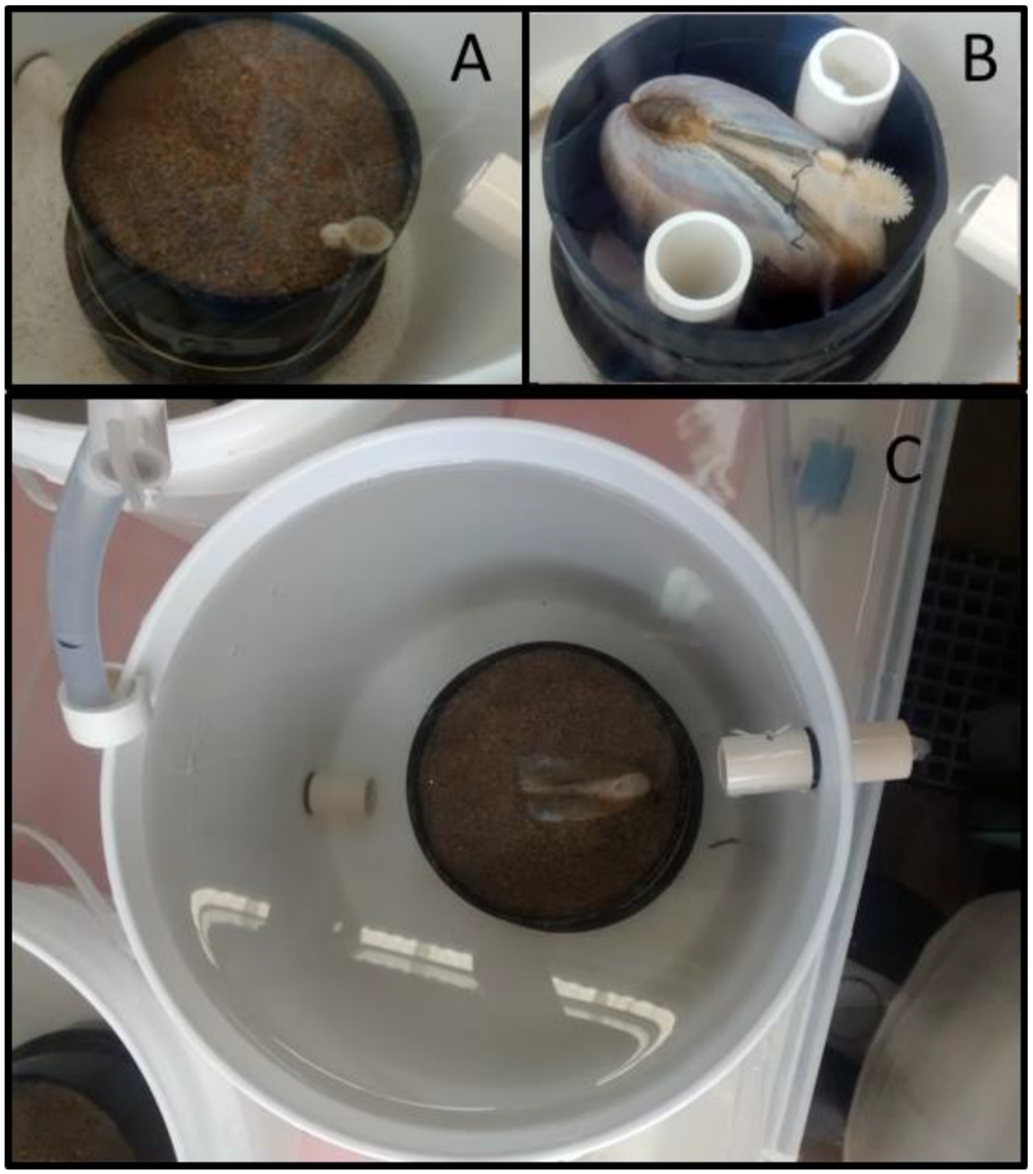
**Experimental open flow experimental units used to evaluate of clearance rates in Pismo clams.** Each unit consisted of a cylindrical 7.6 L white plastic bucket containing a 25 mm diameter ABS (acrylonitrile butadiene styrene) black sieve, where a sand-burrowed (A) or a laterally pressed clam (B) were held in place with their siphons facing upward. Two vertical PVC (polyvinyl chloride) pipes located inside the sieve maintained the lateral pressure of substrate-free clams. The full view of the experimental unit with inflow (bottom) and outflow sections (top) is also shown (C).

**Fig. 2.**
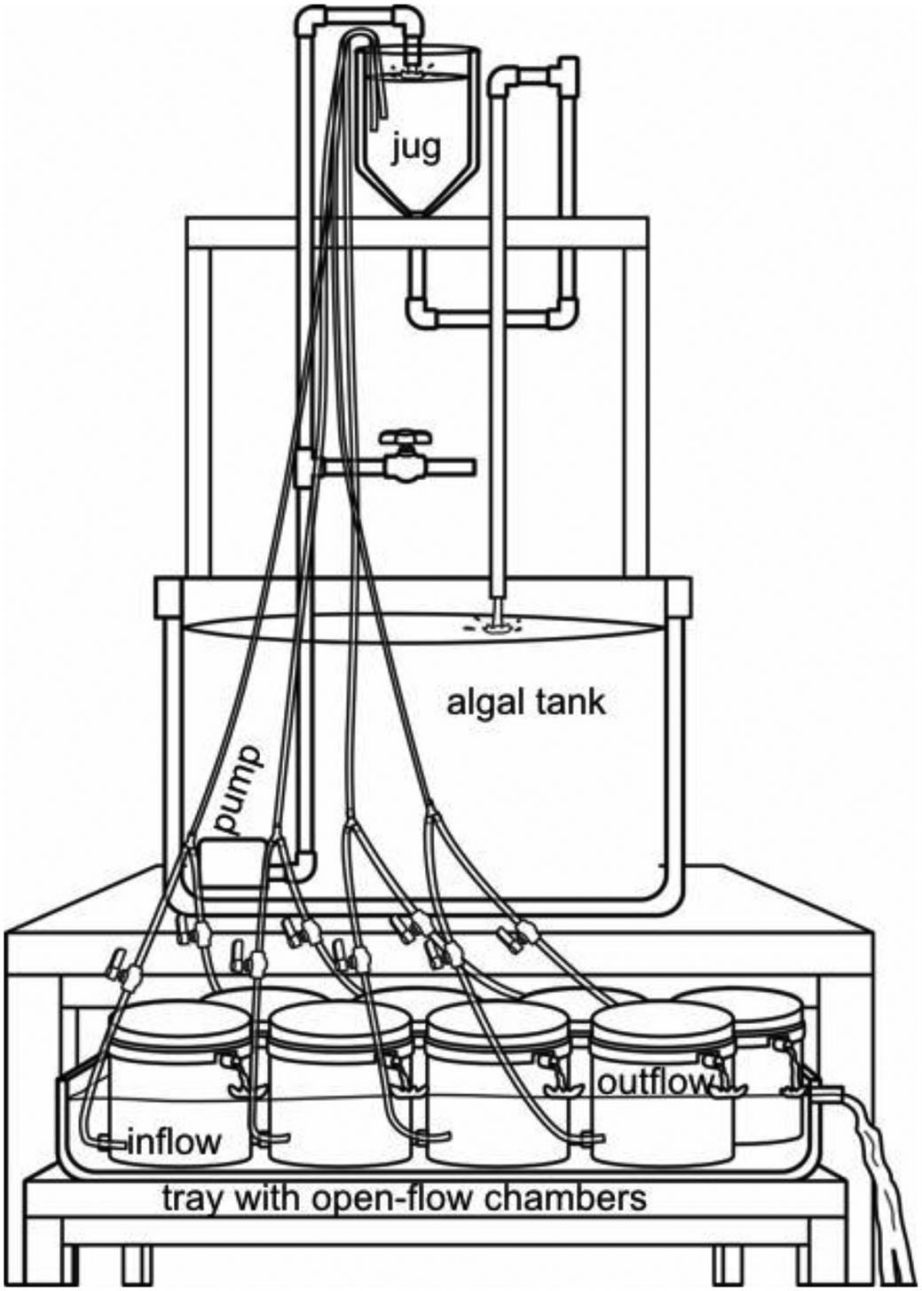
**Diagram (not at scale) of the experimental open flow system.** A main 700 L algal stock tank with a recirculating aquarium pump has an inverted jug with tube level 2 m above the experimental units, where several food-grade plastic tubes delivered the food down to the experimental units by gravity. Flow in each experimental unit is regulated in advance with mini hose valves pressing the plastic tubing.

**Fig. 3.**
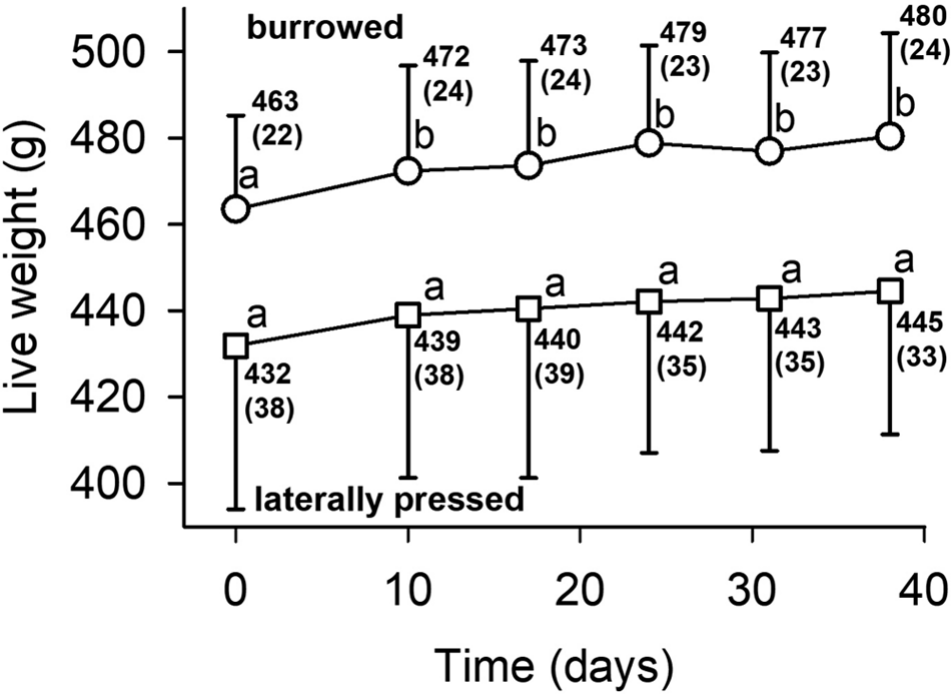
**Temporal changes of the mean live weight of sand-burrowed and laterally-pressed Pismo clams.** Organisms were kept in a recirculating seawater system. Mean values with standard errors (in parenthesis) are shown for each data point. Different superscripts indicate statistical differences inside a given clam group (*P*<0.05).

### Flow effect on clearance rate

**Fig. 4.**
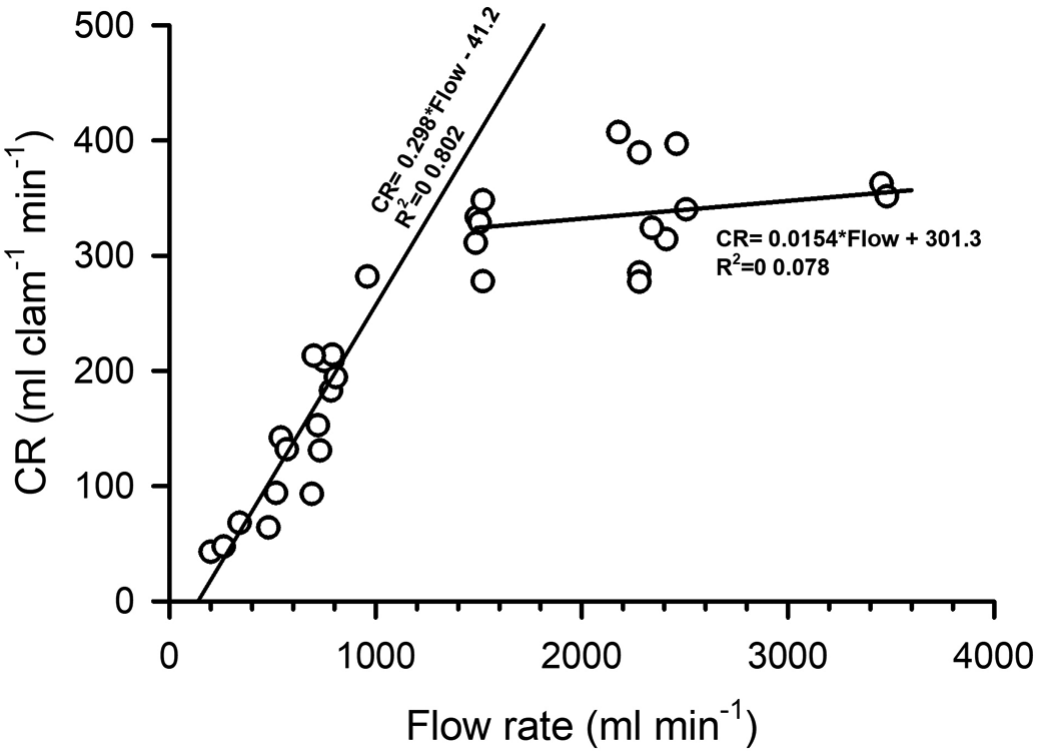
**Mean clearance rates (CR) of Pismo clams as a function of flow.** Linear fit equations and coefficient of determination (R^2^) is shown for clams whose CR increased linearly at flows between 200 and 900 ml and for those exposed to higher flows.

Pismo clams exposed to a concentration from 80–100 cells µl^−1^ and flow rates between 200–3500 ml min^−1^ showed a hyperbolic type of response in their CR, reaching maximal mean values (270–350 ml clam^−1^ min^−1^) at flows from 1000–2500 ml min^−1^ (Fig. 4). Some clams closed their siphons at 3400 ml min^−1^ or higher flows, while those who remained pumping tended to slightly decrease their CR (Fig. 4). Feces produced continuously during assays were ejected through the exhalant siphon as compact rod-like strings that accumulated sideways (Fig. 1B) and no pseudofeces production was evident to the eye. In contrast, feces ejected by clams through the exhalant siphon were conspicuous rod-like strings, rested on top the clam's mantle-shell or sediment and maintained their shape and integrity throughout all assays. An apparent dependency phase between CR and flow was observed in the range from 200–800 ml min^−1^ and coincided with a statistically significant linear regression (*P*<0.001). In contrast, an independency phase was observed at higher flows (Fig. 4), where the linear regression equation was not statistically different from zero (*P*=0.295).

### Temperature effect on clearance rate

**Fig. 5.**
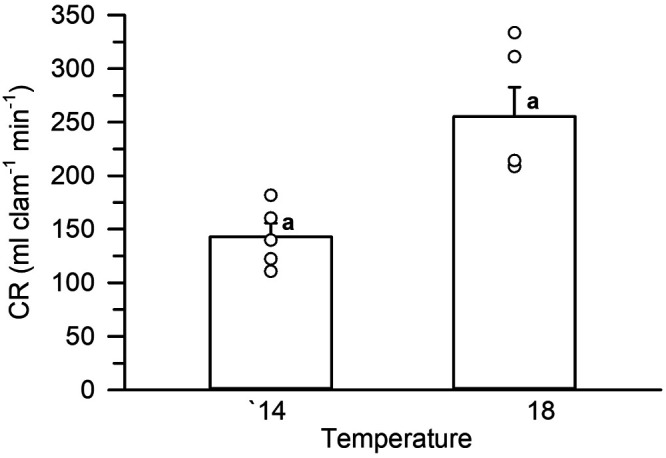
**Mean clearance rates (CR) of Pismo clams exposed at two temperatures.** Bars show the mean CR values exhibited at 14°C and 18°C by the same organisms on different dates. Circles correspond to individual data points obtain for each experimental clam. Similar superscripts indicate no statistical differences among groups (*P*>0.05).

Burrowed organisms exposed to 14°C and flow rates from 1000–1500 ml min^−1^ showed a mean CR value of 142± 13 ml min^−1^ clam^−1^. When the same organisms were exposed to 18°C and same flow pattern, they nearly doubled their CR, reaching a mean value of 255±27 ml min^−1^ clam^−1^ (Fig. 5), which was significantly higer than that at 14°C (paired *t*-test *t*=−3.953, *P*=0.008).

### Clearance rate of burrowed versus laterally pressed clams

**Fig. 6.**
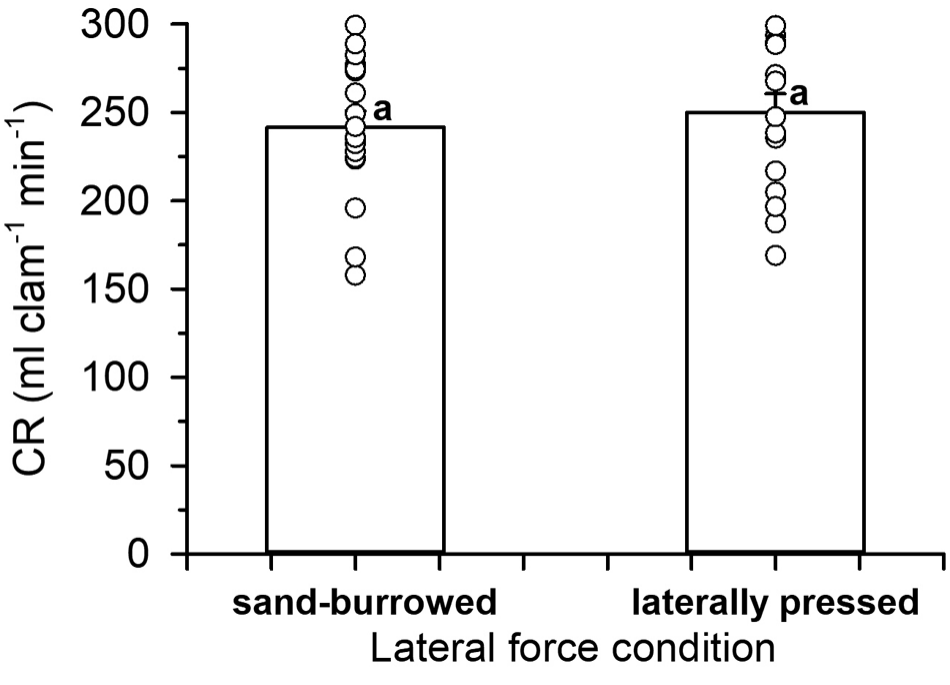
**Mean clearance rates (CR) of burrowed and laterally pressed Pismo clams.** Bars show the mean CR values exhibited by sand-burrowed (A) and laterally-pressed Pismo clams. Circles correspond to individual data points obtain for each experimental clam. Similar superscripts indicate no statistical differences among groups (*P*>0.05).

Both sand-burrowed and laterally pressed, unborrowed Pismo clams behaved similarly inside the experimental units, with their inhalant and exhalant siphons actively pumping the surrounding water and producing fecal threads, respectively. The magnitude of the mean CR (Fig. 6) was strikingly similar in both groups (241±9 and 250±10 ml clam^−1^ min^−1^ for burrowed and laterally pressed organisms, respectively) and no statistical differences were detected among them (two-tailed *t*-test *t*=−0.616, *P*=0.271). For this reason, CR data of burrowed and laterally pressed clams were pooled in subsequent assays and plotted as a single mean for a given test factor.

### Food concentration effect on Clearance Rate

**Fig. 7.**
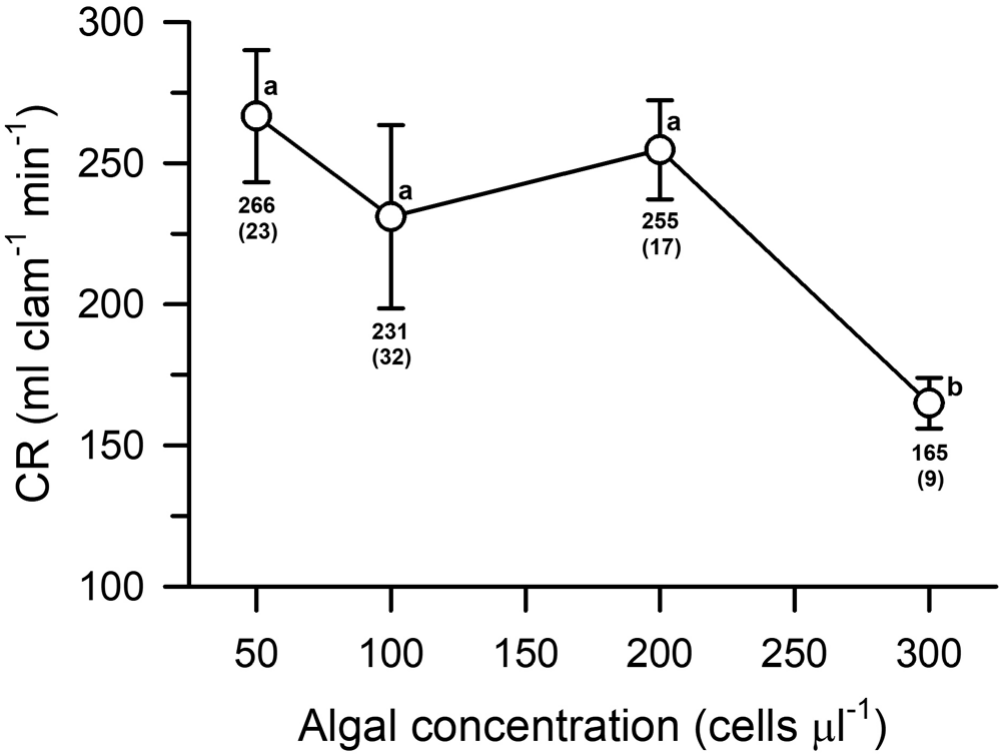
**Mean clearance rates (CR) of Pismo clams as a function of microalgal concentration.** Adult clams were exposed to various cell concentrations of the microalga *Tisochrysis lutea* on different days. Mean values with standard errors (in parenthesis) are shown for each data point. Different superscripts indicate statistical differences (*P*<0.05).

Significant differences were observed in the mean CR of Pismo clams (repeated ANOVA test, *F*=6.845, *P*=0.007) when exposed to various microalgal concentrations on different days (Fig. 7). The mean CR values remained relatively constant over the range from 50–200 cells µl^−1^ (Fig. 7) while significantly lower mean CR (165±9 ml clam^−1^ min) was observed at higher concentrations when compared to the rest of the treatments using Student–Newman–Keuls (SNK) test for multiple comparisons of means (*P*<0.05). Clams exposed to concentrations from 50–200 cells µl^−1^ did not show significant differences of their mean CR (SNK test, *P*>0.05) and their mean CR values ranged from 231–265 ml clam^−1^ min^−1^ (Fig. 7).

## DISCUSSION

This study reveals for the first time that Pismo clams may be maintained healthy in the laboratory without sediment, despite the fact that this short-siphoned species is adapted to live near the surface of sandy sediments frequently exposed to the ocean surf (Coe, 1947) and necessarily need to open and close their valves to move and rebury when needed. Although no reports have been available on the force needed by this species to maintain their valves closed outside the water, preliminary observations carried out in this study made it evident that the adductor muscles of unburied, non-pressed Pismo clams became thinner and weaker with time and the clams eventually died. The same phenomena have been reported elsewhere for the soft-shell clam, *Mya arenaria* (Beal, 2011). Pismo clams have thick shells, a strong hinge ligament and two adductor muscles that are required to open and close the valves (CDFW, 2022). Arranging the organisms in compact rows inside a restricted ventilated tray helped keep their valves pressed and closed while maintaining their freedom to pull out and retract their siphons in the laboratory setup. The lateral force exerted by the clam shells or the PVC tubes when placed in experimental chambers most likely mimicked the horizontal press applied by the sandy sediment in their natural environment.

In the present study, laterally pressed experimental clams apparently maintained overall good health and normal pumping and feeding activity over the period of study, demonstrated by the weight pattern and magnitude of CR exhibited by both the laterally pressed clams and the control, burrowed groups. A parallel study also showed that unburrowed Pismo clams arranged in the way described above and maintained in a recirculating aquaculture system were able to produce ripe gonads and mature gametes (Castro-Acevedo, 2019). In general, bivalves are known to experience a significant lateral force when dug into the sediment, which can be as high as 76 N for razor clams of 14 cm in length (Huang and Tao, 2018). Additionally, the hinge of bivalves acts as a torsional spring that constantly tends to open their shell, and the counteracting low-magnitude force of the adductor muscles maintain the valves closed in the natural environment (Crane and Denny, 2020). The force exerted by the adductor muscles of Pismo clams to keep their shells closed has not been measured to date, but likely this species expends a significant amount of energy to keep their valves shut in the absence of sediment, since they have very strong hinge ligaments and thick shells. Burrowed clams are also expected to need a low force to keep their valves closed under ecological scenarios, since sand sediment contributes with lateral forces that help them keep their valves closed with minimum stress, while allowing the siphons to protrude above the substrate to obtain food. Such is the case of other siphoned suspension feeders with burrowing adaptations to sediment with different grain characteristics (e.g. Zwarts and Wanink, 1989; Ledoux et al., 2023). Previous studies indicate that intertidal/subtidal non-burrowing mussels with relatively thin shells and byssal attachment use a substantial force (∼5–30 N) to keep their valves closed with a minimum closing force (Crane and Denny, 2020). While no current data are available on the force exerted by Pismo clams to maintain their valves closed outside the sediment, outside the water and/or during the process of burrowing, the weight and CR results of the present study showed that the strategy of laterally and continuously pressing the valves of unburrowed Pismo clams resulted appropriate to keep them fully functional in the absence of sediment. This finding may be a valuable tool in the future to maintain Pismo clams in the laboratory and continue elucidating the physiology of this species without the interference of sediment. Keeping adult organisms in the hatchery may also be a very helpful tool for conditioning purposes, eliminating the need to continuously clean and change the sediment.

The results of clearance rates obtained in the first experiment confirmed that the set up consisting of cylindrical experimental buckets containing acrylonitrile butadiene styrene (ABS) sieves with Pismo clams and PVC tubing pressing the organisms were appropriate. Pismo clam valve gaping was then restricted while facilitating full siphon functioning in the flow-through chambers, since characteristic dependency and independency phases were evident when CR was plotted as a function of water flow. The former was directly proportional to flow rate in the range of 200 to 800 ml min^−1^ while CR was clearly independent for higher flows, with values ranging from 250–350 ml min^−1^ clam^−1^. Other studies showed that such a dependency–independency pattern is expected when measuring CR in flow-through chambers with appropriate geometric design (Beiras et al., 1993; Filgueira et al., 2006). Therefore, it is likely that the water-food mixture got fully homogenized before reaching the upper part of the chamber where the Pismo clam siphons were located, near the outflow.

To our knowledge, the present study is the first one to report whole-body physiological rates for Pismo clams. The magnitude of the CRs exhibited by individual organisms of 130 mm shell length (SL) was nearly 5× higher than the values reported previously for 36 mm mussels *Mytilus edulis* (Larsen and Riisgård, 2012). It is also more than an order of magnitude higher than CRs reported for smaller species from the Veneridae family (Table 1). Nevertheless, individual-specific values are difficult to compare with other species because of differences in size, species, experimental and dietary conditions and especially because CR values are mostly reported on a weight-specific basis (see reviews by MacDonald et al., 2006 and Cranford et al., 2011). In the present study, Pismo clams were not sacrificed to obtain dry tissue weight (DTW). However, a previous study carried out by Giese et al. (1967) allows for indirect estimations of their soft tissue+pallial liquid weight (29.6% of the total live weight), flesh weight (65% of the previous value) and dry tissue weight (30% of flesh weight). Considering that Pismo clams from this study (∼460 g live weight) showed a CR of ∼250 ml min^−1^ ind^−1^, this value can be translated into 15 L h^−1^ ind^−1^, 0.17 L h^−1^ g^−1^ flesh weight and 0.6 L h^−1^ g^−1^ dry tissue weight. It is evident that weight-specific CRs exhibited by *Tivella stultorum* were lower than those reported for smaller species from the same family (Table 1, see also Table 5 in Metaxatos and Ignitiades, 2011) but are comparable to maximal CRs reported for *Gomphina veneriformis*, *Chamelea gallina* and *Venerupis pullastra* (Table 1). Weight-specific CR values of Pismo clams were an order of magnitude lower than those reported for the geoduck *Panopea zelandica* (8.1 L h^−1^ g^−1^ DTW, Le et al., 2017), the oyster *Crassostrea virginica* (5 L h^−1^ g^−1^ DTW, La Peyre et al., 2020) and the mussel *Perna viridis* (20 L h^−1^ g^−1^ DTW, McFarland et al., 2013) whose shell length are near 100 mm. In general, it is known that CRs standardized to 1 g DTW of a 60 mm organism range between 2.3 and 3.4 L h^−1^ g^−1^ DTW in scallops, oysters and cockles (Cranford et al., 2011).

**Table 1.**
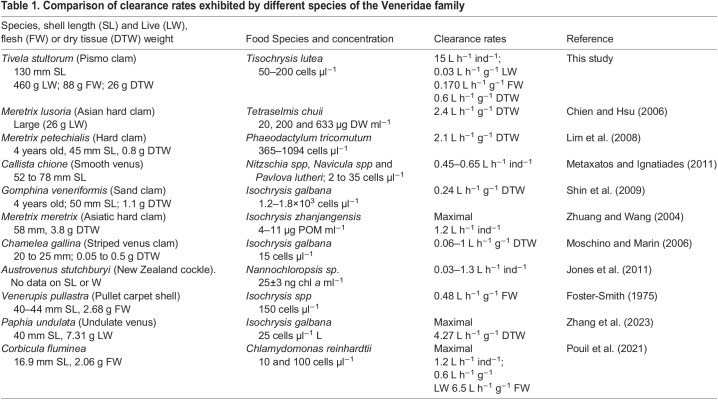
Comparison of clearance rates exhibited by different species of the Veneridae family

The temperature assay showed that Pismo clams nearly doubled their CR with an increase of 4°C. The magnitude of such an increase has also been recorded for other Veneridae species that showed enzyme-like CR pattern to temperature such as *Meretrix petechialis* (up to 3× increase with a 5°C rise) or *G. veneriformis* (up to 8× increase with 5°C rise). Noteworthy, when they approached the lethal temperature, the CR was significantly reduced in both species (Lim et al., 2008; Shin et al., 2009). In contrast, no clear differences of CR were observed in the Asian clam *Corbicula fluminea* (Cyrenidae) exposed to a range of 10–25°C, while a linear-type response was observed in the blue mussel *M. edulis* exposed to acute temperatures ranging 5–20°C after being acclimated to 18°C (Kittner and Riisgård, 2005). From an ecological perspective, Pismo clams live inhabit a coastal zone whose typical surface seawater temperature range from 13–15°C during the fall-winter and 17–20°C in the spring–summer (Alvarez-Borrego and Alvarez-Borrego, 1982), with at least four significant upwelling events during the transition spring–summer and fall–winter (Alvarez-Borrego and Alvarez-Borrego, 1982; Samperio-Ramos et al., 2023). Therefore, the experimental temperatures used in the present study fell within the thermal range experienced by the species, whose gametogenesis begins in early March and intense spawning occurs in San Ramón, Baja California Mexico in July–October (Searcy-Bernal, 1983) when the surface seawater temperature ranges from 17–19°C (Alvarez-Borrego and Alvarez-Borrego, 1982). The spawning peak also coincides with upwelling events and the concomitant increase of primary production and/or particulate organic matter in the area near San Ramón (Mirabal-Gómez et al., 2017; Samperio-Ramos et al., 2023). Therefore, likely Pismo clams are adapted to a significant increase in their clearance and ingestion rate with short-term temperature increases to take advantage of the upwelling events and compensate for the high energy demands of gametogenesis and spawning.

Finally, the CR response of Pismo clams to microalgal concentration appears to indicate that this species has a high plasticity for regulating their CR. Cell densities near or greater than 300 cells µl^−1^ may still reflect a threshold concentration where Pismo clams reduce their pumping rates. In this regard a linear relationship between total gill area and filtering capacity has been already demonstrated in two species of mussels (Jones et al., 1992; Navarro et al., 2011). Thus, 60 mm mussel (*Mytilus chilensis*) with a gill area of ∼3000 mm^2^ exhibited a CR of 2.5 L h^−1^ g^−1^ DW while 20 mm mussels with 700 mm^2^ gill area showed CR of ∼0.7 L h^−1^ g^−1^ DW (Navarro et al., 2011). Both gill area and CR also scaled with an exponent of 2 for shell length in *M. edulis* (Jones et al., 1992). The gill area of experimental adult Pismo clams (130 mm shell length) was not evaluated in the present study, yet gills account for ∼3% of their live weight (Geise et al., 1967) which in turn represents ∼15% of the flesh weight. Therefore, it is anticipated that a 130 mm Pismo clams from the present study have gills with a large area and this may partially explain the high CRs observed even at cell densities of 200 cells µl^−1^ (equivalent to ∼6 mg l^−1^). The threshold concentration values where CR start to decline not only depend physical factors, food concentration or gill area but also on the exposure time of organisms to such concentrations (La Peyre et al., 2020). For example, *Hiatella arctica* (10–50 mm SL) exhibited an exponential decline of CR in the range of 0–27 cells µl^−1^ of *Rhodomonas spp* (Sejr et al., 2004). Threshold concentration of *Isochrysis galbana* were recorded at >10 cells µl^−1^ for 35 mm mussels (Pascoe et al., 2009). Both declining (2 h exposure) and constant CRs (16 h exposure) were recorded in blue mussels (*M. edulis*) when exposed to a range of 1.5–30 cells µl^−1^ of *Phaeodactylum tricornutum* (Riisgård and Randløv, 1981), while highest CRs were observed at 75–200 cells µl^−1^ in *M. edulis*, the cockle *Cerastoderma edule* and the Pullet carpet shell *Venerupis pullastra* after exposing them a range of 10–850 cells µl^−1^ of *P. tricornutum* (Foster-Smith, 1975). A threshold chlorophyll concentration of 26 µg l^−1^ were recorded in mussels exposed to seston containing 2–100 µg chl *a* l^−1^ (Filgueira et al., 2009), while no significant CR effects were found in the American oyster *C. virginica* after chronic exposure to sediment concentrations of 0, 50 and 400 mg l^−1^ (La Peyre et al., 2020). No pseudofeces production was visually observed in the present study even though one of the cell concentrations used was rather high and merit further attention.

In summary, the present study reports for the first-time physiological rates for Pismo clams. The method was developed and validated for measuring reliable clearance rates in Pismo clams in flow-through chambers in the of sediment. The approach used for keeping Pismo clams in the laboratory without sediment resulted in short-term clearance rates and long-term weight changes comparable to those of burrowed organisms. Together, these findings may represent a valuable tool for increasing the study of Pismo clam physiological rates in the laboratory and keeping high numbers of healthy organisms in the hatchery without sediment the interference. Our study shows that adult Pismo clams could maintain clearance rates from 300–400 ml min^−1^ org^−1^ at water flows ranging from 1000–3600 ml min^−1^, while CR was directly dependent on water flow when this variable was <1000 ml min^−1^. A small change of temperature (14–18°C) translated into a large Pismo clam CR change, while this variable was regulated over a wide cell concentration range. These two findings most likely reflect an adaptive Pismo clam response to obtain a maximal food amount when phytoplankton is transiently available in the natural environment, which coincides with transient upwelling events and the species spawning season. However, further studies are needed to gain a better understanding of the physiological adaptation of Pismo clams in nature.

## MATERIALS AND METHODS

### Clam collection and maintenance

Handling, maintenance and sampling protocols of adult Pismo clams followed Mexican guidelines and norms established for good production practices of bivalve molluscs and do not require ethical approval (Calvario-Martínez and Montoya-Rodríguez, 2003). Experimental Pismo clams with shell length between 127–131 mm and 432–463 g live weight were collected in Playa San Ramón, Baja California Mexico in the spring of 2018 and transported in coolers to the Laboratorio de Biotecnología de Moluscos at the Instituto de Investigaciones Oceanológicas, Universidad Autónoma de Baja California in Ensenada, Baja California, Mexico. Shell length (SL) and live weight of clams measured with digital calipers and analytical balance (±0.1 g), respectively and divided in two subgroups. They were transferred to a 700 L fiberglass tank whose bottom was covered by sand where the first subgroup of organisms burrowed. The second subgroup was placed inside a small ventilated-wall plastic crate (59×40×20 cm) without sediment in the same 700 L tank. These clams were arranged side by side on a row along the crate wall, such that their valves were firmly pressing each other (see below). The main 700 L tank was part of a 1500 L recirculating filtered (1 µm) seawater system that included a reservoir tank, biological bubble-bead filter (AST aquaculture systems, Baton Rouge Los Angeles, CA, USA), and heat pump. Changes in live weight of eight clams (four from each subgroup) collected on March 17 was recorded weekly over 5 weeks. The clams collected on April 16 were used for the CR assays listed below. Initial temperature (∼15°C) was increased by 1°C per day and kept at 18°C at which all but one of the experiments were carried out. The flagellate microalga *Tisochrysis lutea* was fed *ad libitum* but after running preliminary experiments, the ration was adjusted on a week-to-week basis considering the number of clams present in the system. Water changes were carried out daily (30%) and sand changed weekly throughout the duration of the experiments.

### Flow-through system for clearance rate assessment

Preliminary experiments showed that Pismo clams kept without substrate were able to eat as usual and produce feces, but their adductor muscles weakened, got thinner, and after several days they were unable to fully close their valves, in such a way that they eventually died. For this reason, a strategy was developed to override this problem by grouping several clams on a row inside the plastic crates. Their valves – oriented anteroposteriorly with siphons and the hinge ligament pointing upwards – were firmly pressing each other. Individual clams used for measuring clearance rates were held inside individual sieves made with acrylonitrile butadiene styrene (ABS) sections (10 cm of diameter, DIA) located inside the buckets. The unborrowed Pismo clams were maintained laterally pressed at the center of the sieve with two PVC pipes (2.5 DIA) held vertically on each side of the clam (Fig. 1B). Parallel CR measurements were carried out on organisms burrowed inside the ABS sieves sections filled with sand (Fig. 1A,C).

Each experimental unit was considered a replicate and consisted of individual ABS pipe sections placed inside 2 gallon (7.6 L) food-grade plastic buckets of 25 cm DIA×24 cm height (Fig. 1A,C). The water and food mixture was delivered with a food-grade 6 mm (internal diameter) plastic tubing that entered through the bucket bottom (Fig. 1C). The discharge tube was located on the opposite and upper side of the bucket (Fig. 1C). All EU were placed inside a plastic 60 L tray that served as water bath (Fig. 2), and temperature was regulated with a 300 W digitally controlled titanium heater and/or chiller. From 4–8 replicates were simultaneously run when measuring CR in any given assay. Before running the assays, microalgal food was diluted with filtered seawater at the desired concentration and placed in a stock 700 L tank located 1 m above the experimental units (Fig. 2). A bottomless inverted 10 L plastic jug was also installed on top of the stock tank with a tube level (Fig. 2), and food was continuously recirculated with an aquarium pump from the bottom of the tank to the jug during the CR assays. Multiple plastic tubes located inside the jug delivered the food down to the experimental units by gravity, and flow was regulated with plastic mini-hose valves (Fig. 2).

### Experiments

#### Assays, sampling and estimation of CR

The first two assays were carried out to test the flow rate and temperature effects on the Pismo clam CRs using burrowed organisms. Two additional assays were implemented in parallel with burrowed (positive control) and laterally pressed clams to test the effects of valve compression (flow 1500 ml min^−1^, cell concentration 100 cells µl^−1^) and food concentration (flow 1500 ml min^−1^, 50,000 to 300,000 cells ml^−1^) on the clam's CR using a flow of 1500 ml min^−1^. A flow rate of 1500 ml min^−1^. In all cases, the experimental organisms were drawn from the main recirculating system, transferred to the experimental flow-through units and stabilized for 2 h at the appropriate food concentration and water flow. Food and water were replenished in the stock tank before starting the assays. Control (blank) experimental units without clams were also included in each assay to correct microalgal sedimentation, which in general was negligible (<2%). Water–food samples of 50 ml were withdrawn from the bucket inflow and outflow at five different moments: initial (0, 0.5, 1, 1.5 and 2 h). Microalgal concentration was quantified immediately after sampling with a Multisizer 3 Coulter Counter (Beckman Coulter). In the present study, we assumed that the experimental chamber had the optimal geometry for determining CR using the flow-through method. Thus, individual CRs were estimated for each sampling interval for clams present in the experimental units using the modified equation suggested by Filgueira et al. (2006) and Larsen and Riisgård (2012) when water inflow enters at the bottom of the chamber and outflow at the top on the opposite side:
(1)

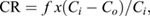
where CR=clearance rate (ml min^−1^); *f*=flow through entering the experimental unit; C_i_ and C_o_ are the incoming and outflowing food concentrations, respectively.

The CR calculations for a given 30 min interval were only performed when C_o_ values decreased from 10–30% with respect to C_i_. The CR estimated for each time interval was averaged over the whole 2-h duration of each assay in each experimental unit, and the mean value was computed for the total number of units.

#### Flow effects

The assays to determine the flow rate effect on CR were carried out using only sand-burrowed organisms. A total of 5–8 experimental units with one organism each were repeatedly used every week over a period of one month to quantify CR. On any given date, the organisms were drawn from the main system, transferred to experimental flow-through units and randomly assigned to any of the following flow rates used on different dates: 500–800; 800–2100; 2300–3500; 700–2500, and 200–1500 ml min^−1^. The organisms were stabilized for 2 h to get used to the experimental conditions (food concentration of 90–100 cells µl^−1^), and after this period the stocking food tank was refilled and sampling performed as indicated in the previous section.

#### Temperature

Two assays were carried out with five organisms to test the temperature effect on Pismo clam CRs. Six burrowed clams kept at 14°C were drawn out from the main recirculating system and transferred to the experimental units containing sediment; CR was assessed at flow rates from 1000–1500 ml min^−1^ and 14°C following the protocol described above. Experimental clams were returned to the main recirculation system at the end of the assay, and temperature was increased by 1 C° per day until reaching 18°C. The organisms remained at 18°C for 2 additional days. After this period, the organisms were drawn out again from the main recirculation system and CRs assessed at 18°C using the same flow rate range.

#### Burrowed versus valve-compressed unburrowed clams

The assay was carried out at 18°C using clams burrowed in sand (positive control) contained by ABS sieves and clams whose shell was laterally compressed with PVC tubing located inside the ABS sieves (Fig. 1). Pumping activity (CR) was quantified in parallel to test if lateral shell compression affected Pismo clams in the absence of sand substrate. Between 6 and 8 experimental units of each group were run in parallel using a flow rate of 1500 ml min^−1^ and 100 cells µl^−1^. After the stabilization period, assays were carried out as indicated above. Assays were carried out twice on different dates using the same organisms.

#### Microalgal concentration

Four assays were carried out on different dates on the same organisms to test the effect of food concentration eon the CR of Pismo clams. Three to four burrowed and laterally pressed clams were used in parallel in four different assays using the following food concentrations (one food concentration per assay): 50, 100, 200 and 300 cells µl^−1^. Assays were carried out at 18°C and a flow rate of 1500 ml min^−1^.

### Statistics

Linear regression analysis of CR as a function of flow rate as used on two groups of data: those obtained with flows ranging from 200–1000 ml min^−1^ and flows >1000 ml min^−1^. Analysis of variance (ANOVA) was applied to test time-dependent changes in live weight, followed by pairwise multiple comparisons of means with the Student–Newman–Keuls (SNK) method. Assays to test the effect of cell concentration on the mean CR of Pismo clams were carried out on the same individuals at different dates. For this reason, repeated-measures analysis of variance (RMANOVA) was used to compare their mean CRs, followed SNK comparisons. A paired *t*-test was used to compare temperature effects while a Student’s *t*-test was applied to compare the mean CR of burrowed versus laterally pressed Pismo clams.

## Diversity and inclusion statement

The authors believe in equal opportunity in all areas of professional development and every person is unique in their own way. We aim to create an open, inclusive, and equal environment in which every person has the opportunity to succeed. Participants developing and publishing this research is an example of such practice.
